# Demographic and Clinical Features of Patients with Metastatic Breast Cancer: A Retrospective Multicenter Registry Study of the Turkish Oncology Group

**DOI:** 10.3390/cancers15061667

**Published:** 2023-03-08

**Authors:** Izzet Dogan, Sercan Aksoy, Burcu Cakar, Gul Basaran, Ozlem Ercelep, Nil Molinas Mandel, Taner Korkmaz, Erhan Gokmen, Cem Sener, Adnan Aydiner, Pinar Saip, Yesim Eralp

**Affiliations:** 1 Department of Medical Oncology, Institute of Oncology, Istanbul University, Istanbul 34093, Turkey; izzet.dogan@istanbul.edu.tr (I.D.);; 2 Department of Medical Oncology, Hacettepe University Cancer Institute, Ankara 06100, Turkey; 3 Department of Medical Oncology, Faculty of Medicine, Ege University, Izmir 35100, Turkey; 4 Department of Medical Oncology, Acibadem University, Altunizade Acibadem Hospital, Istanbul 34662, Turkey; 5 Department of Medical Oncology, Faculty of Medicine, Marmara University, Istanbul 34722, Turkey; 6 Department of Medical Oncology, Koç University Amerikan Hospital, Istanbul 34010, Turkey; 7 Department of Medical Oncology, Acibadem University, Maslak Acibadem Hospital, Istanbul 34457, Turkey; 8 Incidence Medical Research and Biostatistics Consultancy Services, Istanbul 34440, Turkey; 9 Research Institute of Senology, Acıbadem University, Maslak Acıbadem Hospital, Istanbul 34457, Turkey

**Keywords:** breast cancer, metastasis, treatment, human epidermal growth factor receptor 2, hormone receptor, registries

## Abstract

**Simple Summary:**

Despite advances in treatment generated by clinical trials in metastatic breast cancer (MBC), their impact on routine daily practice and the reflection of the outcome within the community remains unclear. This study evaluates time-related differences in treatment patterns and outcome in a real-world patient population with MBC over a ten-year timeframe. Except for the HER2+ subgroup, which showed a significant survival benefit with the incorporation of novel agents, we failed to identify significant variations in outcomes for the remaining subgroups. A consistent feature we observed was the challenge in treating TNBC, which had the worst prognosis in both time-related cohorts. Elucidation of biologic characteristics to identify novel treatment options remains an unmet need to improve outcomes in TNBC. The favorable survival attained with routine endocrine agents in the luminal A subgroup suggests that barriers in access to CDK inhibitors may not have a negative impact on the outcome in subgroups of hormone receptor-positive patients, constituting an appealing strategy for communities with limited resources.

**Abstract:**

This multicenter registry study aims to analyze time-related changes in the treatment patterns and outcome of patients with metastatic breast cancer (MBC) over a ten-year period. Correlations between demographic, prognostic variables and survival outcomes were carried out in database aggregates consisting of cohorts based on disease presentation (recurrent vs. de novo) and the diagnosis date of MBC (Cohort I: patient diagnosed between January 2010 and December 2014; and Cohort II: between January 2015 and December 2019). Out of 1382 patients analyzed, 52.3% patients had recurrent disease, with an increased frequency over time (47.9% in Cohort I vs. 56.1% in Cohort II, *p* < 0.001). In recurrent patients, 38.4% (*n* = 277) relapsed within two years from initial diagnosis, among which triple-negative BC (TNBC) was the most frequent (51.7%). Median overall survival (OS) was 51.0 (48.0–55.0) months for all patients, which was similar across both cohorts. HER2+ subtype had the highest OS among subgroups (HER2+ vs. HR+ vs. TNBC; 57 vs. 52 vs. 27 months, *p* < 0.001), and the dnMBC group showed a better outcome than recMBC (53 vs. 47 months, *p* = 0.013). Despite the lack of CDK inhibitors, luminal A patients receiving endocrine therapy had a favorable outcome (70 months), constituting an appealing approach with limited resources. The only survival improvement during the timeframe was observed in HER2+ dnMBC patients (3-year OS Cohort I: 62% vs. Cohort II: 84.7%, *p* = 0.009). The incorporation of targeted agents within standard treatment has improved the outcome in HER2+ MBC patients over time. Nevertheless, despite advances in early diagnosis and treatment, the prognosis of patients with TNBC remains poor, highlighting the need for more effective treatment options.

## 1. Introduction

According to the Globocan registry, breast cancer (BC) is the most frequent cancer type among women in Turkey, with 24,175 new cases diagnosed in 2020, comprising 23.9% of all female cancers nationwide [1]. Based on the 2017 Turkish registry database, approximately 10% of all new patients present with metastatic disease annually, remaining relatively stable over the last decade [2]. Nevertheless, despite the similar incidence on a global scale, the estimated 5.7% mortality rate compares favorably with the global mortality rate reaching 15.5%, reflecting widespread adoption of modern diagnostic and therapeutic techniques in the management of patients diagnosed with breast cancer in Turkey [1,3]. Guidelines and reimbursement strategies for the diagnosis and treatment of oncology patients are determined through discussions held by the scientific and financial committees established under Order by the Turkish Ministry of Health. These national guidelines, consisting of evidence-based practice patterns and sequential treatment options, are implemented by the Social Security System to cover all healthcare expenses of Turkish citizens throughout the country. In accordance with these guidelines reflecting most of the modern treatment approaches in the higher Human Development Index countries, metastatic breast cancer patients have access to most targeted agents as well as cytotoxic and endocrine agents, which are updated regularly based on scientific evidence as well as fiscal and monetary policies of the time.

Although the prognosis of specific subtypes of metastatic BC (MBC) patients seems to have improved over the last decade, the outcome is highly variable based on differences in presentation, patient-related factors, genomic landscape of the disease, as well as disparities in healthcare and access to novel medications [4,5,6,7]. Advances in diagnostic techniques and increased awareness, especially in communities with a strong health infrastructure and high income, have resulted in a lower incidence of de novo metastatic presentation at initial diagnosis, with incidence rates declining from around 25–28% at the turn of the century to 6–9% in the past decade [8,9]. This shift in metastatic patterns may have affected prognosis over time, as metastatic disease following treatment for early-stage disease has been universally associated with a poor outcome. The shorter survival of recurrent MBC (recMBC) has been linked to several adverse prognostic factors, including a higher incidence of challenging subtypes such as triple-negative BC (TNBC) or the selection of resistant clones within histologic subgroups [10,11,12]. In fact, a retrospective U.S. cohort study encompassing a period of three decades extending from 1990 to 2020 has revealed a decrease in the incidence of metastatic progression from early-stage disease, whereas the incidence of de novo MBC (dnMBC) remained relatively constant. In concordance with the expected differences in outcomes, a reverse trend in 5-year cancer-specific survival (CSS) over time was noted, showing an approximately two-fold improvement in the de novo cohort from 28 to 55%, and a deterioration in recMBC from 23% to 13% [12].

Elucidating prognostic variances over time is critical for improving our understanding of the impact of modern treatment approaches in distinct pathologic subgroups and providing further insight into the evolving biology of metastatic patterns. Therefore, this large multicenter registry study was planned to examine survival differences in MBC over the last decade in a qualified real-life setting.

## 2. Materials and Methods

### 2.1. Study Design

The Turkish Oncology Group MBC was a multicenter retrospective registry study that aimed to collect the data of adult MBC patients diagnosed between 1 January 2010 and 31 December 2019 at seven tertiary oncology clinics in Turkey. The participating sites, which were identified based on patient volume, academic background, as well as dedication to breast cancer diagnosis and treatment, are academic-based public and private oncology centers known to deliver high-quality healthcare in accordance with globally accepted consensus guidelines. Since all investigators who were invited to participate agreed to contribute, there was no bias in regard to data collection among centers included in the study. Collectively, the database reflects real-world practice in both private and public-based comprehensive academic oncology centers from the three most populated cities comprising 28% of the Turkish population, providing a unique opportunity to evaluate changes in contemporary treatment patterns and outcomes over the analyzed period. Correlations between demographic, prognostic variables and survival outcomes were carried out in database aggregates consisting of cohorts based on disease presentation (recurrent vs. de novo) and the diagnosis date of MBC (Cohort I: patient diagnosed between January 2010 and December 2014; and Cohort II: between January 2015 and December 2019). The primary objective was to assess the impact of changes in utilization of modern treatment options on the outcome of various prognostic subgroups. Secondary endpoints included characterization of metastatic presentation patterns (recurrent vs. de novo) within the specified timeframe and outcomes. The study protocol was approved by the Acıbadem Mehmet Ali Aydınlar University Medical Research Ethics Committee (Approval no and date: 2020-23/35, 5 November 2020). Patients who had given consent for the use of medical records were included in the study.

### 2.2. Patients and Statistical Analysis

Adult patients aged 18 years or older who were diagnosed with MBC as reported by the investigators were included in this database. De novo disease was defined as MBC diagnosed concurrently or within 3 months of initial BC diagnosis. Initial pathologic diagnosis and treatment details of patients presenting with recMBC were collected from patient charts and reports provided by the investigator. Non-visceral disease was defined as skeletal, distant lymphatic or soft tissue metastasis. The number of metastatic sites were defined as the number of visceral systems involved, or in the case of non-visceral disease, as the number of distinct sites which were not in juxtaposition to an index lesion. Pathologic subgroups of recMBC were preferably based on metastatic site biopsies where available. Hormone-responsive (HR+) disease was defined as membranous estrogen (ER) or progesterone (PR) receptor expression in at least 1% of tumor cells. Luminal A was defined as ER ≥10% (+), PR ≥20% (+), Her2 (−) and Ki 67 < 20%. Patients were classified as luminal B disease if the tumors were PR < 20%, or Ki67 > 20%, or grade 3. Human epidermal growth factor receptor 2 (HER2) assessment was carried out according to the ASCO CAP 2018 guidelines by the pathology departments of each participating center. Tumors expressing ER or PR and HER2 were classified as luminal B-HER2+ tumors. TNBC was defined as tumors not expressing ER, PR or HER2.

Treatment details were recorded from patient charts, and first-line treatment was described as initial therapy following diagnosis of metastatic disease until progression. Endpoints were defined as: progression-free survival (PFS): time from metastatic diagnosis to first progression or death, whichever occurs first; overall survival (OS): time from metastatic diagnosis to death from any cause; and disease-free interval (DFI): defined as the time from initial diagnosis in the early disease setting to first recurrence.

Treatment patterns were compared descriptively between dnMBC and recMBC cohorts for the whole group and separately for each time period. Fisher’s exact test or Chi-square test and the Mann–Whitney U-test were used to compare baseline patient and disease characteristics for categorical and continuous variables, respectively. Survival outcomes were estimated using the Kaplan–Meier product-limit method and compared within each subgroup by the log-rank test. Each endpoint was corrected for established prognostic factors. Hazard ratios (HRs) and their 95% confidence intervals (CIs) were estimated using the Cox regression analysis. Factors that were statistically significant in the univariate model were included in the multivariate model. Analyses were performed using SPSS version 23.0 (IBM Corp. Released 2015. IBM SPSS Statistics for Windows, Version 23.0. Armonk, NY, USA) and MedCalc statistical software version 12.7.0.0 (MedCalc Software, Ostend, Belgium). *p* values less than or equal to 5% were considered significant.

## 3. Results

The whole group recorded in the database included 1381 patients, with 641 and 740 patients analyzed in Cohorts I (January 2010–December 2014) and II (January 2015–December 2019), respectively. The median age of the whole patient group was 48 (range 17–91), comprising 755 (62.1%) HR+, 333 (27.4%) HER2+ and 128 (10.5%) TN patients. There were 342 patients (25%) younger than 40 years. Despite the significant shift towards private-based institutions after 2015 (17.6% vs. 30.3%, *p* < 0.001), significantly more patients were treated at community-based academic centers (*n* = 1044, 75%) as compared to private-based academic centers in the whole group (*n* = 337; 25%; *p* < 0.001). There was no difference in the distribution of relevant prognostic factors, including age (*p* = 0.117), stage at presentation (for recMBC only; *p* = 0.84), histology (*p* = 0.42), number of metastatic sites (*p* = 0.21) and use of ablative/local therapy in either cohort (17.5 vs. 15.5% in Cohort I vs. II; *p* = 0.33). At presentation, there were more patients with bone-only disease in the HR+ group (*n* = 417; 62.2%) as compared to HER2+ (*n* = 122; 18.2%) and TN (*n* = 38; 5.7%) subtypes (*p* < 0.001), with a similar distribution in each cohort. There was a numeric increase in the incidence of CNS involvement over time in the HER2+ (Cohort I: *n* = 13; 7.1%; Cohort II: *n* = 18; 12.0%) and the TN subgroups (Cohort I: *n* = 8; 11.8%; Cohort II: *n* = 11; 18.3%) as compared to the HR+ subtype (Cohort I: *n* = 14; 4.5% vs. Cohort II: *n* = 19; 4.2%) (*p* = NS). Furthermore, there was a trend for a higher ratio of very young patients with MBC aged < 40 in Cohort II among HER2+ (32 vs. 20.2%, *p* < 0.001) and TN (23.3 vs. 19.1%, *p* = NS) patients. Demographic characteristics in Cohorts I and II are summarized in Table 1.

### 3.1. Recurrent MBC

Out of 1381 patients analyzed, 52.3% (*n* = 722) of patients had recurrent disease, with an increased frequency over time (47.9% in Cohort I vs. 56.1% in Cohort II, *p* < 0.001). The median age of the patients was 46, ranging between 20 and 81. There was a higher incidence of premenopausal patients in the recMBC group as compared to de novo patients (*p* < 0.001). Forty six percent (*n* = 337) presented with bone-only disease, whereas 316 (43.8%) presented with visceral involvement and 69 (9.6%) with CNS metastasis. There were significantly more patients with HR+ disease (*n* = 404; 55.9%), as compared to HER2+ (*n* = 144; 19.9%) and TN groups (*n* = 87; 12.04%) (*p* < 0.001). Nevertheless, the majority of TN patients presented with recurrent disease as compared to dnMBC in the whole patient population (*n* = 87 vs. 41; 67.9% vs. 32.1%; *p* = 0.109). Time-dependent variations within the entire recMBC group regarding subgroups revealed a significant increase in the ratio of HR+ patients in Cohort II (*n* = 251; 60.5%) vs. Cohort I (*n* = 153; 49.8%) (*p* = 0.004), with an even distribution in luminal A (Cohort I: *n* = 59, 19.2% vs. Cohort II: *n* = 99, 23.9%; *p* = 0.14) vs. luminal B disease (Cohort I: *n* = 94, 30.6% vs. Cohort II: *n* = 152, 36.6%; *p* = 0.09). There was an opposite trend over time noted for HER2+ (Cohort II: *n* = 77, 18.6% vs. Cohort I: *n* = 67, 21.8%; *p* = 0.277), as well as TN patients (Cohort II: *n* = 45, 10.8% vs. Cohort I: *n* = 42, 13.7%; *p* = 0.247) (Table 2).

In regard to DFI, 38.4% (*n* = 277) had relapsed within two years from initial diagnosis, comprising mostly the HR+ subtype (*n* = 154; 55.5%), followed by the HER2+ (*n* = 62, 22.4%) and TN (*n* = 45; 16.2%) subgroups. There were significantly more patients who relapsed within 24 months in Cohort II (*n* = 174; 62.8%) as compared to Cohort I (*n* = 103; 37.2%; *p* = 0.02). When analyzed separately within pathologic subtypes, the ratio of rapid progressors was the highest among the TNBC group (51.7%) (vs. the HER2+ (43.1%) and HR+ (38.1%; luminal A = 36.1% vs. luminal B = 39.4%) groups (*p* = 0.056)). Time-related changes in disease characteristics within each pathologic subgroup are summarized in Table 2.

### 3.2. De Novo MBC

There were 659 patients (47.7%) who presented with dnMBC in the entire cohort, consisting of 351 (53.2%) with HR+ disease, 189 (28.7%) with HER2+ and 41 (6.2%) with TN MBC. Despite a decreasing frequency over time (63.4% in Cohort I vs. 48.7% in Cohort II, *p* = 0.007), the HER2+ subtype was the largest group among all pathological subgroups presenting with de novo disease. The median age of the whole group was 50, ranging between 17 and 91. There was a higher ratio of patients with skeletal metastasis in the HR+ subgroup (56.4%) as compared to HER2+ (38.1%) and TN (24.4%) patients, and an opposite trend for visceral metastasis in each subgroup, respectively (40.7% vs. 58.7% vs. 68.3%; *p* < 0.001). The ratio of patients presenting with CNS involvement was highest in TN patients (7.3%) vs. HR+ (2.8%) and HER2+ (3.2%) subgroups (*p* = 0.313). Disease characteristics regarding metastatic presentation (recMBC vs. dnMBC) are summarized in Table 2.

### 3.3. Treatment Patterns

A significantly higher ratio of patients with HR+ disease received first-line chemotherapy (CT) in Cohort I (*n* = 148; 48.2%) vs. Cohort II (*n* = 172; 38.9%; *p* = 0.01), with an opposite trend for endocrine therapy (ET) (Cohort I (*n* = 118; 38.4%) vs. Cohort II (*n* = 194; 43.9%; *p* = 0.14)). Nevertheless, there was no change in trends to deliver CT as a first-line treatment to dnMBC in either cohort (Cohort I: *n* = 79; 50.3% vs. Cohort II: *n* = 89; 45.9%; *9* = 0.41) as compared to recMBC patients, who were less likely to receive front-line CT in Cohort II (Cohort I: *n* = 69; 46.0% vs. Cohort II: *n* = 83; 33.5%; *p* = 0.013). A minority of patients in Cohort II were treated with ET + CDK inhibitors as a first-line therapy following regulatory approval in 2019 (*n* = 28; 6.3%). In the HER2+ subgroup, there was a similar ratio of patients receiving standard first-line CT + HER2 blockade over time (Cohort I vs. Cohort II, 39.0% vs. 35.5%; respectively). In Cohort II, 27 (18.0%) patients were treated with CT + dual HER2 blockade with trastuzumab and pertuzumab, which was more frequently utilized in de novo (*n* = 18; 24.7%) vs. recurrent patients (*n* = 9; 11.7%; *p* = 0.06). There was a higher ratio of patients with TNBC who received platin-based front-line CT in Cohort II (*n* = 19; 32.8%) vs. Cohort I (*n* = 14; 22.2%; *p* = 0.273). Immunotherapy and CT combination was given to seven patients in Cohort II (12.1%) (dnMBC: *n* = 3; 20% vs. recMBC: *n* = 4; 9.3%; *p* = NS). A summary of front-line therapy for all subgroups within each cohort is given in Table 3.

### 3.4. Outcomes

Median PFS for all patients at initial treatment for metastatic disease was 18.0 (17.0–19.0) months, while significant variances were identified within pathologic subtypes (HR+ vs. HER2+ vs. TNBC; 19 vs. 18 vs. 10 months, *p* < 0.001). After a median follow-up period of 36 (0–142) months and 778 (56.3%) events, the median OS was 51.0 (48.0–55.0) months for all patients, with the TN subtype having the worst OS (HER2+ vs. HR+ vs. TNBC; 57 vs. 52 vs. 27 months, *p* < 0.001).

**Figure 1:**
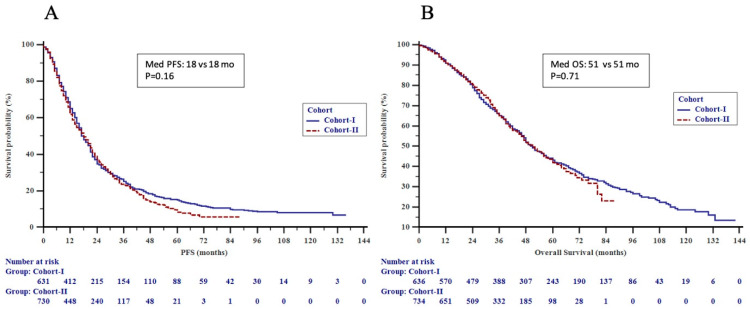
(**A**) Progression-free survival (PFS) by time cohorts and (**B**) overall survival (OS) by time cohorts in the patients.

As for the primary endpoint, there was no significant difference in the outcome among patients in Cohorts I vs. II (51 vs. 51 months, *p* = NS) (Figure 1A,B). Nevertheless, time-related changes in outcomes were noted within HER2+ and HR+ subgroups dependent on metastatic presentation, as described in detail below.

**Figure 2:**
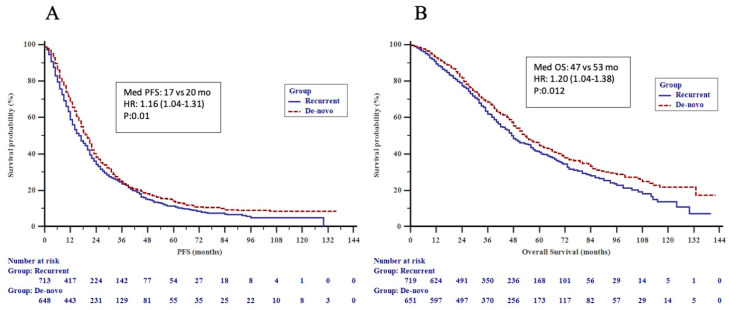
(**A**) Progression-free survival (PFS) by metastatic pattern and (**B**) overall survival (OS) by metastatic pattern in the patients.

We observed a significant difference in PFS (HR 1.16, 95% CI 1.04–1.31, *p* = 0.01) and OS (HR 1.20, 95% CI 1.04–1.38, *p* = 0.01) in dnMBC as compared to recMBC (Figure 2A,B). When recurrent patients were analyzed with respect to DFI, the TNBC subgroup showed a significantly higher OS in DFI ≥ 24 vs. DFI < 24 months (36 vs. 20 months; *p* = 0.043). Older age at presentation (≥50), recurrent disease, visceral and CNS metastatic involvement, ≥3 metastatic sites at presentation and luminal B, and HER2+ and TNBC subtypes (vs. luminal A) were significantly associated with a poorer outcome by univariate analysis. Older age (≥50), luminal B and TNBC subtypes (vs. luminal A), visceral and CNS metastatic involvement remained as independent predictors of poor OS by multivariate analysis (Table 4). When recurrent patients were analyzed separately, older age, luminal B and TNBC subtypes (vs. luminal A), stage III at initial diagnosis (vs. stage I and II), and visceral metastasis were identified as independent prognostic factors for a poorer overall survival (Table 5).

### 3.5. HER2+ Subgroup

**Figure 3:**
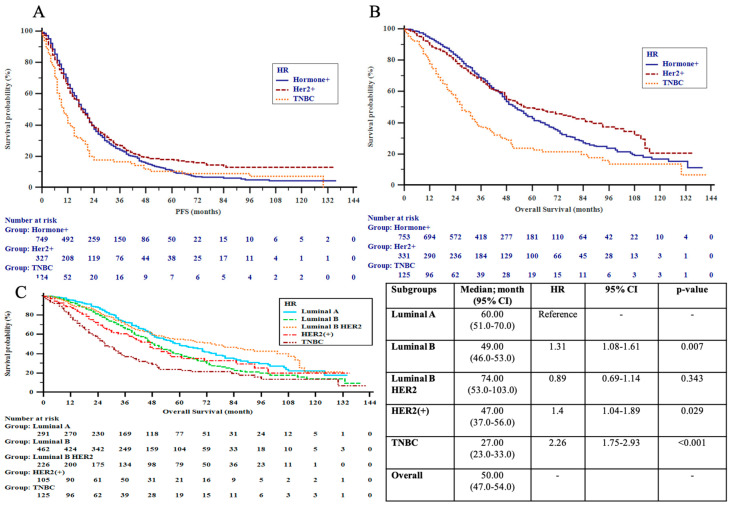
(**A**) Progression-free survival (PFS) by pathologic subgroups; (**B**) overall survival (OS) by pathologic subgroups; (**C**) overall survival (OS) by extended pathologic subgroups in the patients. HER2 = human epidermal growth factor receptor 2; TNBC = triple-negative breast cancer.

Following conditional approval of use in visceral dnMBC in 2016, dual-HER2 blockade with trastuzumab and pertuzumab was more frequently used in Cohort II compared to Cohort I (*p* < 0.001), leading to substantial improvements in outcomes. Survival analysis revealed significant benefits in the de novo group in alignment with the approval indication for dual blockade (Cohort I vs. II; 3-year OS: 62.0% vs. 84.7%, *p* = 0.009), especially noted in those with visceral metastatic presentation (59.4% vs. 83.4%, *p* = 0.03), luminal B-HER2+ disease (61.2% vs. 89.2%, *p* = 0.013) and younger age < 40 years (40.0% vs. 94.7%, *p* = 0.009). The improvement in median OS in the de novo HER2+ group was linked to the favorable outcome in the luminal B-HER2+ subgroup, which showed a 3-year OS rate of 89.2% vs. 61.2% in Cohorts I and II, respectively (*p* = 0.013) (Table 6a,b and Figure 3A–C).

### 3.6. HR+ Subgroup

Despite the insignificant numeric improvement in PFS and OS in dnMBC patients, the outcomes of HR+ patients remained similar over the time points analyzed, reflecting the similar practice patterns in the use of first-line treatment and barriers to access CDK inhibitors. Patients in Cohort II with HR+ recMBC, who were more likely to receive first-line endocrine therapy than the previous 5-year period, showed similar OS and PFS, despite the higher incidence of unfavorable prognostic factors such as luminal B disease (60.6%) and a higher ratio of endocrine-resistant patients (Cohort I 32% vs. Cohort II 41.8%; *p* = 0.049). In Cohort II, the prognoses of recurrent luminal B patients were significantly worse as compared to recurrent luminal A patients (median OS: 44 vs. 76 months, *p* = 0.012).

When both time-related cohorts were combined, patients with luminal A who received ET as first-line treatment had a significant improvement in OS as compared to those who were treated with CT (70 months (95% CI 52–88) vs. 48 months (95%CI 35–61), respectively; *p* = 0.008). Luminal B patients had a numeric improvement in OS with first-line ET vs. CT (56 months (95% CI 46–66) vs. 46 months (95% CI 41–51); *p* = 0.135). There was no difference noted in PFS achieved with either treatment modality in both luminal A and B pathologic subtypes (Table 7).

### 3.7. TN Subgroup

TN patients had the poorest outcome among all patients analyzed, with no significant improvement over time. Unexpectedly, recurrent patients in the latter cohort had a significantly worse PFS (7 vs. 15 months, *p* = 0.023) and OS (20 vs. 42 months, *p* = 0.005), most probably due to unfavorable prognostic factors such as a higher incidence of early progressors within two years after initial diagnosis (55.6% vs. 47.6%) and an increased ratio of CNS metastasis at presentation (18.3% vs. 11.8%).

There was a non-significant numeric increase in survival over time in the de novo group (26 vs. 22 months), 20% of whom had access to immunotherapy and 66.7% of whom received conventional non-platin-based chemotherapy in the first-line setting. When patients with ER < 10% (*n* = 11) were added to the de novo TN group, the outcomes remained similar (29 vs. 22 months, *p* = 0.421).

## 4. Discussion

In this retrospective multicenter cohort, we observed significant differences in metastatic presentation and outcome among histologic subgroups of MBC patients over the analyzed period. In contrast to existing data from large registry studies, our cohort included a high ratio of recurrent patients which increased over time from 48% to 56% [12,13]. Furthermore, we also observed a significant time-dependent increase in the incidence of refractory patients who developed metastatic disease within two years of early-stage BC treatment, consisting mainly of HR+ and TN subgroups. In fact, the proportion of TN patients showed an incremental increase among de novo (6.2%), recurrent patients with DFI > 24 months (9.4%) versus DFI < 24 months (16%), whereas the ratio of HR+ patients remained constant, accounting for the poor biologic behavior in refractory recurrent patients consistent with previous reports [13,14,15]. Nevertheless, the high incidence of dnMBC (43.9%) in Cohort II exceeds the previously reported ratios of de novo presentation, ranging between 28 and 30% among all MBC patients [16,17]. We also observed a higher proportion of de novo presentation among the entire HER2+ subgroup (48.7%), which is in line with existing data reporting that 37.5–49.8% of HER2+ MBC present with de novo disease [13,18,19,20,21].

Although there was no difference among both time-related cohorts based on age, 42% of patients diagnosed with MBC were premenopausal and there was a higher ratio of patients younger than 40 among HER2+ and TN subgroups. In fact, population-based studies have indicated a skewed age distribution towards a younger population with unfavorable prognosis over the last three decades. There has been a consistent increase noted in annual hazards of advanced stage at diagnosis in patients aged 25–39 among all race and ethnic groups analyzed, with a higher incidence of TN and HER2+ subgroups which were unaccounted for by clinical or genomic features [22,23]. Nevertheless, our findings suggested that younger age was independently associated with a favorable outcome, consistent with data from a recent study focusing on young patients with dnMBC. In this study, improved survival was noted in all subgroups except those with TNBC, indicating that variances in tumor biology might account for survival disparities [24]. In fact, a biomarker analysis of a retrospective case–control cohort has shown differential gene expression of de novo versus recurrent MBC, a finding which needs validation by further studies [19].

The median survival of the whole cohort over the analyzed period was 51 months. Although patients with de novo mBC had a significantly longer OS than those with recurrent disease by univariate analysis (53 vs. 47 mo; *p*: 0.013), the presentation pattern was not shown to be independently associated with the outcome (Table 4). Our findings compare favorably with previous registry studies which have reported median OS ranging between 22 and 37 months, with wide variations among pathologic subgroups [7,12,13,19,25,26,27]. Nevertheless, our findings indicate that de novo presentation may not be an independent prognostic factor per se. The favorable outcome may be associated with several confounders such as a lower tumor burden due to advances in diagnostic techniques, impact of age, histology, lack of resistance ensued by previous treatment pressure or a distinct biologic behavior independent of clinicopathologic factors as discussed by several studies [14,28,29]. Nevertheless, similar outcomes have been observed in recurrent patients with a long DFI. These observations suggest that there may be other contributing factors in the evolution of metastatic disease. In fact, outcomes of control arms from more recent phase III trials have repeatedly yielded superior results in comparison to data from registration studies, suggesting that time-related advances in diagnostic modalities and access to optimized health care systems could play a role in reported survival disparities [28,30,31,32,33]. With the caveat of making cross-trial comparisons, it is not possible to draw firm conclusions on time-related variances in survival. Although translational studies from large-scale prospective studies provide valuable information on spatial biologic characteristics of distinct subgroups, future prospects to address temporal variances in outcomes require a multi-faceted approach combining standardized modern health care with in-depth genomic monitoring of micrometastatic disease.

Although there was no difference noted in patient characteristics and outcomes between the two time-related cohorts, the only difference in survival over time was observed in the HER2+ subgroup, which reached significance in de novo luminal B-HER2+ patients treated over the last five-year period. Despite bearing an unfavorable patient profile enriched in a younger population with CNS involvement, the improvement in outcome in the HER2+ dnMBC most likely reflects the higher rates of access to combined trastuzumab and pertuzumab after 2015. Our results are in parallel with several registry data showing a significant outcome difference in patients with de novo as compared to recurrent HER2+ MBC which have reported superior survival rates only in the HER2+ subgroup [18,34,35,36,37]. A striking finding in our cohort was the favorable prognosis in the luminal B-HER2+ subgroup as compared to all pathologic subtypes, which has been consistently observed by others, reflecting the use of sequential endocrine therapy following chemotherapy and HER2 blockade in routine clinical practice [9,38,39]. In the absence of robust randomized data, clinical practice patterns favoring this approach have evolved through large-scale prospective registry data demonstrating improved outcomes with the addition of ET following completion of CT and HER2 blockade as compared to CT and HER2 targeting alone [40].

A consistent observation over the analyzed period was the poor survival in the TN subgroup, which has been determined as an independent prognostic factor on overall mortality in our cohort, as well as many others [8,20,26,27,41].

In concordance with contemporary community-based studies that have failed to reflect the significant survival benefits demonstrated by clinical trials, we did not observe significant variations in outcomes neither within the entire HR+ group (52 months), nor when broken down into luminal A (60 months) and luminal B (49 months) subgroups [27,35]. Although we collected data from private-based academic centers, a formal comparison of outcomes was not carried out, as this endpoint is not within the scope of the present analysis due to an inherent risk of potential bias. In general, the private sector is estimated to provide healthcare for approximately 30% of oncology patients nationwide, which is in line with our private-based cohort comprising 25% of the whole patient population. Although all centers included in this registry were chosen based on their ability to deliver optimal, standardized and high-quality healthcare, we have to acknowledge that there may be barriers in receipt of cancer care in academic-designated public centers which have been burdened by a growing patient volume, exceeding their capacity to provide timely and supportive care. Furthermore, a lack of optimized social and physical support, as well as difficulties in access to modern treatment options or enrollment in clinical trials, may account for disparities in health care in the general community setting. Therefore, it requires the countrywide collaboration of cancer centers with the Ministry of Health to identify barriers for accessible and value-based care, which will provide guidance in developing policies to implement equitable health care throughout the nation.

Nevertheless, recurrent luminal A patients had a significantly longer OS compared to luminal B patients in Cohort II (76 vs. 44 months, *p* = 0.012), which could be attributable to a time-related shift in first-line management of HR+ MBC from a higher ratio of CT use in Cohort I (CT 46% vs. ET 37%) to ET in Cohort II (ET 44% vs. CT 33%, *p* = 0.008). The inappropriate preference for CT as the initial therapy in our patient population contradicts recent guidelines and real-world experience that have reported more frequent use of ET for up to 70% of HR+ patients [13,26,27,35,42]. In fact, a contemporary Turkish observational study including 758 HR+ MBC patients treated between 2019 and 2020 reported a significant increase in ET use with 70% of patients receiving ET and CDK inhibitors as first-line therapy and a subsequent decline in first-line CT use from 49% to 20% following regulatory approval, which was associated with a significant improvement in PFS [43]. Nevertheless, despite strong evidence for improved OS with CDK inhibitors in the first-line setting reaching 64 months, the favorable OS ranging between 49 and 76 months in our luminal B and A subgroups without access to contemporary endocrine targeted agents may provide an appealing option in limited resource settings [44].

Our study has many inherent limitations due to the retrospective nature of a registry database lacking information on comorbid conditions, menopausal status, family histories and genomic factors, all of which may have confounded the results. Data obtained from the heterogenous patient population cannot be extrapolated to the whole nation, especially in underserved areas. Most importantly, subtype classifications for most recurrent patients were based on initial pathology reports at initial diagnosis rather than repeated biopsies at metastatic presentation. This may have confounded outcomes in some histologic subgroups as they are more likely to include patients with poorer prognosis, especially in those with early recurrences. We were not able to assess the impact of novel therapies such as CDK inhibitors or immunotherapy since they were not approved for use at that time. Furthermore, data on time-on-treatment for switch maintenance ET or HER2 blockade could not be captured from patient files, which would provide valuable data on the impact of subsequent therapies for each prognostic subgroup.

Nevertheless, the main strengths of this study that should be mentioned are the collaborative efforts of tertiary academic centers providing high-quality pathologic data and standardized management within national limits. The data generated from this registry study reflects real-life practice patterns in both private and social security reimbursed systems while minimizing the impact of variances in routine diagnostic and management strategies. Furthermore, the patient population belongs to the three most populated cities with a high domestic migration rate, which represents national characteristics of MBC to a large extent.

## 5. Conclusions

In conclusion, our findings provide further proof that improved survival in MBC is associated with advances in treatment as observed especially in luminal B-HER2+ patients over the analyzed period. In fact, the unprecedented success of anti-HER2 therapies has affirmed that clinically relevant outcomes from trials adopted in routine practice can revolutionize the prognosis of a subgroup, highlighting the relevance of targeting biology. Furthermore, a consistent feature we observed was the challenge in treating TNBC, which was identified as the worst prognostic subgroup without any correlation with clinicopathologic confounders. Elucidation of biologic characteristics to identify novel treatment options remains an unmet need to improve outcomes in TNBC. Nevertheless, with increasing demand from the community to have access to newer-generation novel agents, the financial burden of cancer care has risen dramatically over the past decade. Emerging evidence suggests that real-world data provide relevant information on challenges to implement evolving therapeutic options in routine practice and the impact of increasing costs in widening social gaps and disparities in access to optimal health care [45]. Given the inherent heterogeneity of the analyzed cohort and complexities of decision making to treat MBC, we acknowledge the limitations of our data. However, the findings of this study may provide unique insights into the dynamics of practice patterns and outcomes, which may be used by healthcare authorities to identify whether the adoption of modern treatment options has improved survival and to shed light on future interventions to enhance quality of care.

## Figures and Tables

**Table 1:** Patient and treatment-related characteristics in all patients and subgroups.

Characteristics	All Patient (*n* = 1381)			HR+ (*n* = 755)			HER2+ (*n* = 333)			TNBC (*n* = 128)		
	Cohort I	Cohort II	*p*	Cohort I	Cohort II	*p*	Cohort I	Cohort II	*p*	Cohort I	Cohort II	*p*
**n (%)**	641 (46.4)	740 (53.6)		310 (41.1)	445 (58.9)		183 (55)	150 (45)		68 (53.1)	60 (46.9)	
**Treatment center**			**<0.001**			**<0.001**			**0.002**			**0.021**
Private-based	113 (17.6)	224 (30.3)		55 (17.7)	138 (31)		30 (16.4)	46 (30.7)		9 (13.2)	19 (31.7)	
Community-based	528 (82.4)	516 (69.7)		255 (82.3)	307 (69)		153 (83.6)	104 (69.3)		59 (86.8)	41 (68.3)	
**Disease status**			**<0.001**			0.056			**0.007**			0.158
Recurrent	307 (47.9)	415 (56.1)		153 (49.4)	251 (56.4)		67 (36.6)	77 (51.3)		42 (61.8)	45 (75)	
De novo	334 (52.1)	325 (43.9)		157 (50.6)	194 (43.6)		116 (63.4)	73 (48.7)		26 (38.2)	15 (25)	
**Age group, median (range)**	48 (17–84)	49 (20–91)	0.117	47 (17–84)	50 (23–82)	**0.009**	47 (20–80)	46 (25–91)	0.852	50 (24–83)	46 (29–81)	0.176
<40	167 (26.1)	175 (23.7)	**0.010**	99 (31.9)	100 (22.5)	**<0.001**	37 (20.2)	48 (32)	**0.014**	13 (19.1)	14 (23.3)	0.453
40–45	104 (16.2)	136 (18.4)		45 (14.5)	80 (18)		40 (21.9)	24 (16)		11 (16.2)	14 (23.3)	
45–50	104 (16.2)	81 (11.0)		45 (14.5)	47 (10.6)		32 (17.5)	12 (8)		12 (17.6)	11 (18.3)	
50–70	226 (35.3)	309 (41.8)		99 (31.9)	197 (44.3)		67 (36.6)	57 (38)		27 (39.7)	20 (33.3)	
>70	34 (6.1)	31 (4.7)		22 (7.1)	21 (4.7)		7 (3.8)	9 (6)		5 (7.4)	1 (1.7)	
**Stage at diagnosis**	n = 262	n = 357	0.844	n = 135	n = 226	0.740	n = 61	n = 65	0.542	n = 32	n = 37	0.793
I	29 (11.1)	45 (12.6)		14 (10.4)	28 (12.4)		6 (9.8)	10 (15.4)		2 (6.3)	4 (10.8)	
II	98 (37.4)	131 (36.7)		49 (36.3)	86 (38.1)		19 (31.1)	22 (33.8)		15 (46.9)	16 (43.2)	
III	135 (51.5)	181 (50.7)		72 (53.3)	112 (49.6)		36 (59)	33 (50.8)		15 (46.9)	17 (45.9)	
**Histology ^†^**	n = 286	n = 381	0.416	n = 148	n = 242	**0.014**	n = 65	n = 74	0.368	n = 37	n = 40	0.117
IDC	243 (85.0)	305 (80.1)		126 (85.1)	185 (76.4)		56 (86.2)	66 (9.27)		33 (89.2)	33 (82.5)	
ILC	22 (7.7)	41 (10.8)		8 (5.4)	35 (14.5)		5 (7.7)	2 (2.7)		3 (8.1)	1 (2.5)	
Mixed	18 (6.3)	29 (7.6)		14 (9.5)	18 (7.4)		2 (3.1)	5 (6.8)		0 (0)	5 (12.5)	
Other	3 (1.0)	6 (1.6)		0 (0)	4 (1.7)		2 (3.1)	1 (1.4)		1 (2.7)	1 (2.5)	
**ER receptor level**	n = 516	n = 629	**<0.001**	n = 291	n = 436	**0.043**	n = 160	n = 137	**0.012**	n = 57	n = 51	NA
Negative	120 (23.3)	100 (15.9)		3 (1)	2 (0.5)		58 (36.3)	45 (32.8)		57 (100)	51 (100)	
1–9%	9 (1.7)	13 (2.1)		5 (1.7)	6 (1.4)		4 (2.5)	6 (4.4)		0 (0)	0 (0)	
10–20%	27 (5.2)	24 (3.8)		14 (4.8)	11 (2.5)		13 (8.1)	12 (8.8)		0 (0)	0 (0)	
21–50%	70 (13.6)	43 (6.8)		37 (12.7)	33 (7.6)		32 (20)	10 (7.3)		0 (0)	0 (0)	
>50%	290 (56.2)	449 (71.4)		232 (79.7)	384 (88.1)		53 (33.1)	64 (46.7)		0 (0)	0 (0)	
**DFI from EBC diagnosis**	n = 307	n = 415	**0.022**	n = 153	n = 251	**0.049**	n = 67	n = 77	0.775	n = 42	n = 45	0.459
<24 month	103 (33.6)	174 (41.9)		49 (32.0)	105 (41.8)		28 (41.8)	34 (44.2)		20 (47.6)	25 (55.6)	
≥24 month	204 (66.4)	241 (58.1)		104 (68.0)	146 (58.2)		39 (58.2)	43 (55.8)		22 (52.4)	20 (44.4)	
**Number of metastatic sites at initial metastatic presentation**	n = 641	n = 737	0.211	n = 310	n = 443	0.675			0.124			0.139
≤3	544 (84.9)	607 (82.4)		257 (82.9)	362 (81.7)		160 (87.4)	122 (81.3)		65 (95.6)	52 (86.7)	
>3	97 (15.1)	130 (17.6)		53 (17.1)	81 (18.3)		23 (12.6)	28 (18.7)		3 (4.4)	8 (13.3)	
**Use local ablative treatment/surgery for oligometastatic disease**			0.334			0.912			0.142			1.000
No	529 (82.5)	625 (84.5)		254 (81.9)	366 (82.2)		149 (81.4)	131 (87.3)		57 (83.8)	50 (83.3)	
Yes	112 (17.5)	115 (15.5)		56 (18.1)	79 (17.8)		34 (18.6)	19 (12.7)		11 (16.2)	10 (16.7)	
**Sites of specific metastatic sites at initial metastatic presentation**			0.090			0.708			0.066			**0.037**
Bone-only	292 (45.6)	378 (51.1)		168 (54.2)	249 (56)		67 (36.6)	55 (36.7)		16 (23.5)	22 (36.7)	
Visceral	310 (48.4)	312 (42.2)		128 (41.3)	177 (39.8)		103 (56.3)	77 (51.3)		44 (64.7)	27 (45)	
CNS	23 (3.6)	35 (4.7)		7 (2.3)	13 (2.9)		6 (3.3)	15 (10)		7 (10.3)	5 (8.3)	
Visceral + CNS	16 (2.5)	15 (2.0)		7 (2.3)	6 (1.3)		7 (3.8)	3 (2)		1 (1.5)	6 (10)	

^†^ Data of 45 patients with unknown histology were excluded from the analysis. CNS = central nervous system; DFI = disease-free interval; EBC = early breast cancer; ER = estrogen receptor; HER2+ = human epidermal growth factor receptor 2 positive; HR+ = hormone-responsive disease; IDC = invasive ductal carcinoma; ILC = invasive lobular carcinoma; TNBC = triple-negative breast cancer.

**Table 2:** Patient characteristics by cohorts and metastatic pattern.

	Cohort I			Cohort II		
	Recurrent (n = 307)	De Novo (n = 334)	*p*-Value	Recurrent (n = 415)	De Novo (n = 325)	*p*-Value
**Age, median (range)**	46 (22–80)	49 (17–84)	**<0.001**	47 (20–81)	52 (23–91)	**<0.001**
**Pathology subtypes, n (%)**	n = 262	n = 299	**<0.001**	n = 373	n = 282	**0.007**
HR+	153 (58.4)	157 (52.5)		251 (67.3)	194 (68.8)	
HER2+	67 (25.6)	116 (38.8)		77 (20.6)	73 (25.9)	
TNBC	42 (16.0)	26 (8.7)		45 (12.1)	15 (5.3)	
**HR+ subgroups, n (%)**	n = 153	n = 157	0.335	n = 251	n = 194	0.198
Luminal A	59 (38.6)	69 (43.9)		99 (39.4)	65 (33.5)	
Luminal B	94 (61.4)	88 (56.1)		152 (60.6)	129 (66.5)	
**Stage at early disease n (%)**	n = 262		NA	n = 357		NA
I	29 (11.1)	NA		45 (12.6)	NA	
II	98 (37.4)	NA		131 (36.7)	NA	
III	135 (51.5)	NA		181 (50.7)	NA	
**Metastatic Sites, n (%)**	n:298	n:327	**<0.001**	n:406	n:319	**0.003**
Bone-only	137 (44.6)	155 (46.4)		200 (48.2)	178 (54.8)	
Visceral	140 (45.6)	170 (50.9)		176 (42.4)	136 (41.8)	
CNS	21 (6.8)	2 (0.6)		30 (7.2)	5 (1.5)	

CNS = central nervous system; HER2+ = human epidermal growth factor receptor 2 positive; HR+ = hormone-responsive disease; TNBC = triple-negative breast cancer.

**Table 3:** Time-related changes in first-line treatment patterns.

	Cohort I				Cohort II			
Subgroups	De Novo n (%)	Recurrent n (%)	*p*-Value		De Novo n (%)	Recurrent n (%)	*p*-Value	
**HR+**	157 (51.1)	150 (48.9)		0.363	194 (43.9)	248 (56.1)		**0.014**
CT	79 (50.3)	69 (46.0)	0.449		89 (45.9)	83 (33.5)	**0.008**	
ET	62 (39.5)	56 (37.3)	0.698		84 (43.3)	110 (44.4)	0.824	
ET + CDKi	1 (0.6)	0 (0)	1.000		8 (4.1)	20 (8.1)	0.136	
CT + ET	4 (2.5)	7 (4.7)	0.489		7 (3.6)	20 (8.1)	0.082	
Other	11 (7.0)	18 (12.0)	0.194		6 (3.1)	16 (6.0)	0.164	
**HER2+**	116 (63.4)	67 (36.6)		0.349	73 (48.7)	77 (51.3)		0.131
CT + trastuzumab	50 (43.1)	21 (31.3)	0.116		12 (16.4)	15 (19.5)	0.786	
CT + dual blockade	1 (0.9)	0 (0)	1.000		18 (24.7)	9 (11.7)	0.064	
ET + trastuzumab	10 (8.6)	8 (11.9)	0.639		1 (1.4)	6 (7.8)	0.117	
ET + dual blockade	0 (0)	0 (0)	NA		1 (1.4)	1 (1.3)	1.000	
Other	55 (47.4)	38 (56.7)	0.225		41 (56.2)	46 (59.7)	0.657	
**TNBC**	26 (41.3)	37 (58.7)		0.167	15 (24.2)	43 (75.8)		0.542
CT (non-platin)	19 (73.1)	19 (51.4)	0.141		6 (40.0)	15 (34.9)	0.966	
CT (with platin)	3 (11.5)	11 (29.7)	0.161		3 (20.0)	16 (37.2)	0.340	
CT + Immunotherapy	0 (0)	0 (0)	NA		3 (20.0)	4 (9.3)	0.360	
Other	4 (15.4)	7 (18.9)	1.000		3 (20.0)	8 (18.6)	1.000	

CDKi = cyclin-dependent kinase inhibitors; CT = chemotherapy; ET = endocrine therapy; HER2+ = human epidermal growth factor receptor 2 positive; HR+ = hormone-responsive disease; TNBC = triple-negative breast cancer.

**Table 4:** Univariate and multivariate analysis for overall survival in the whole cohort with metastatic breast cancer.

	Univariate Analysis		Multivariate Analysis	
	Hazard Ratio (CI 95%)	*p* Value	Hazard Ratio (CI 95%)	*p* Value
**Age** (<50 vs. ≥50)	1.25 (1.08–1.44)	**0.002**	1.25 (1.07–1.45)	**0.005**
**Metastatic pattern** (De novo vs. Recurrent)	1.19 (1.04–1.37)	**0.013**	1.14 (0.97–1.33)	0.100
**Cohorts** (Cohort II vs. Cohort I)	0.97 (0.84–1.13)	0.712		
**Histopathological subtype**				
Luminal A	Reference		Reference	
Luminal B	1.31 (1.08–1.61)	**0.007**	1.29 (1.06–1.58)	**0.013**
Luminal B-HER2+	0.89 (0.69–1.14)	0.343	0.85 (0.66–1.09)	0.188
HER2+	1.40 (1.04–1.89)	**0.029**	1.27 (0.94–1.73)	0.122
TNBC	2.26 (1.75–2.93)	**<0.001**	2.04 (1.57–2.66)	**<0.001**
**Number of metastatic sites** (≤3 vs. >3)	1.23 (1.03–1.49)	**0.023**	1.28 (1.05–1.59)	**0.013**
**Visceral metastasis** (No vs. Yes)	1.33 (1.15–1.53)	**<0.001**	1.37 (1.17–1.61)	**<0.001**
**CNS metastasis** (No vs. Yes)	1.56 (1.19–2.04)	**0.001**	1.95 (1.46–2.62)	**<0.001**
**Use local ablative treatment/surgery** (Yes vs. No.)	0.95 (0.79–1.15)	0.635		

CNS = central nervous system; HER2+ = human epidermal growth factor receptor 2 positive; TNBC = triple-negative breast cancer. Initial variants are analyzed as the referent variable. Multivariate analysis model *p* value ≤ 0.001.

**Table 5:** Univariate and multivariate analysis for overall survival in the recurrent patients with metastatic breast cancer.

	Univariate Analysis		Multivariate Analysis	
	Hazard Ratio (CI 95%)	*p* Value	Hazard Ratio (CI 95%)	*p* Value
**Age** (<50 vs. ≥50)	1.41 (1.16–1.72)	**<0.001**	1.38 (1.10–1.74)	**0.005**
**Cohorts** (Cohort II vs. Cohort I)	0.97 (0.79–1.19)	0.778		
**Histopathological subtype**				
Luminal A	Reference		Reference	
Luminal B	1.37 (1.04–1.80)	**0.027**	1.39 (1.04–1.87)	**0.026**
Luminal B-HER2+	0.86 (0.59–1.24)	0.412	0.97 (0.65–1.44)	0.865
HER2+	1.76 (1.15–2.70)	**0.010**	1.50 (0.96–2.34)	0.077
TNBC	2.13 (1.53–2.98)	**<0.001**	2.17 (1.49–3.15)	**<0.001**
**DFI** (≥24 months vs. <24 months)	1.05 (0.86–1.28)	0.608		
**Stage at presentation** (I + II vs. III)	1.36 (1.10–1.68)	**0.004**	1.52 (1.21–1.90)	**<0.001**
**Number of metastatic sites** (≤3 vs. >3)	1.32 (1.00–1.72)	0.054		
**Visceral metastasis** (No vs. Yes)	1.47 (1.21–1.78)	**<0.001**	1.53 (1.22–1.92)	**<0.001**
**CNS metastasis** (No vs. Yes)	1.35 (0.98–1.85)	0.068		
**Use local ablative treatment/surgery** (Yes vs. No)	0.81 (0.62–1.06)	0.126		

CNS = central nervous system; DFI = disease-free interval; HER2+ = human epidermal growth factor receptor 2 positive; TNBC = triple-negative breast cancer. Multivariate analysis model *p* value ≤ 0.001.

**Table 6:** Overall survival (OS) and progression-free survival (PFS) in recurrent and de novo patients within each time cohort.

**a. In All Pathologic Subgroups**									
**Pathology Subtypes**		**Recurrent MBC**				**De Novo MBC**			
		**N Events/** **Total N**	**Cohort I**	**Cohort II**	***p***	**N Events/Total N**	**Cohort I**	**Cohort II**	***p***
**HR+**	OS	225/403	109/153	116/250	0.681	199/351	112/157	87/194	0.121
	Median (95% Cl), months		49 (43–55)	48 (40–56)			57 (46–68)	52 (47–57)	
	PFS	352/404	141/153	211/251	0.308	298/351	144/157	154/194	0.424
	Median (95% Cl), months		18 (15–21)	17 (13–21)			21 (18–24)	20 (18–22)	
**Luminal A**	OS	76/157	41/59	35/98	0.551	72/134	44/69	28/65	0.195
	Median (95% Cl), months		53 (40–66)	76 (49–103)			70 (52–88)	53 (43–63)	
	PFS	139/158	54/59	85/99	0.710	110/134	62/69	48/65	0.551
	Median (95% Cl), months		17 (14–20)	22 (16–28)			22 (15–29)	20 (17–23)	
**Luminal B**	OS	149/246	68/94	81/152	0.346	127/217	68/88	59/129	0.409
	Median (95% Cl), months		48 (40–56)	44 (39–49)			52 (45–59)	49 (43–55)	
	PFS	213/246	87/94	126/152	0.255	188/217	82/88	106/129	0.668
	Median (95% Cl), months		21 (16–26)	15 (11–19)			17 (14–20)	21 (18–24)	
**HER2+**	OS	73/144	43/67	30/77	0.340	95/189	74/116	21/73	**0.009**
	1-year survival %		86.4%	88.2%			89.6%	97.3%	
	2-year survival %		73.1%	79.8%			74.6%	91.4%	
	3-year survival %		54.3%	68.2%			62.0%	84.7%	
	PFS	84/136	40/64	44/72	0.671	63/186	42/114	21/72	**0.037**
	Median (95% Cl), months		12 (9–15)	20 (16–24)			17 (15–19)	29 (19–39)	
**TNBC**	OS	64/87	31/42	33/45	**0.005**	31/40	21/26	10/14	0.731
	Median (95% Cl), months		42 (32–52)	20 (14–26)			22 (11–33)	26 (13–39)	
	PFS	49/77	19/35	30/42	**0.023**	24/37	15/23	9/14	0.741
	Median (95% Cl), months		15 (11–19)	7 (5–9)			9 (6–12)	8 (6–10)	
**b. In HR+ and HER2+ subgroups**									
**Pathology Subtypes**		**Recurrent MBC**				**De novo MBC**			
		**N Events/Total N**	**Cohort I**	**Cohort II**	***p***	**N Events/Total N**	**Cohort I**	**Cohort II**	***p***
**Luminal A**									
	OS	76/157	41/59	35/98	0.551	72/134	44/69	28/65	0.195
	Median (95% Cl), months		53 (40–66)	76 (49–103)			70 (52–88)	53 (43–63)	
**Luminal B**									
	OS	149/246	68/94	81/152	0.346	127/217	68/88	59/129	0.409
	Median (95% Cl), months		48 (40–56)	44 (39–49)			52 (45–59)	49 (43–55)	
			*p* = 0.444	***p* = 0.012**			*p* = 0.104	*p* = 0.591	
**Luminal B-HER2+**									
	OS	44/94	27/45	17/49	0.606	64/132	51/81	13/51	**0.013**
	3-year survival %		64.8%	75.9%			61.2%	89.2%	
**HR–/HER2+**									
	OS	29/50	16/22	13/28	0.197	31/57	23/35	8/22	0.378
	Median (95% Cl), months or 3-year survival %		24 (13–35)	47 (32–62)			63.9%	74.2%	

HR+ = hormone-responsive disease; HER2+ = human epidermal growth factor receptor 2 positive; MBC = metastatic breast cancer; OS = overall survival; PFS = progression-free survival; TNBC = triple-negative breast cancer.

**Table 7:** Treatment-related survival outcomes in luminal A and B subgroups.

	n (%)	PFS (Month)	*p*	OS (Month)	*p*
**Overall**					
**Luminal A**					
CT-ET	111 (45.5)	20	0.849	48	**0.008**
ET	133 (54.5)	20		70	
**Luminal B**					
CT-ET	209 (53.9)	17	0.711	46	0.135
ET	179 (46.1)	18		56	
**Cohort I**					
**Luminal A**					
CT-ET	60 (53.1)	19	0.293	49	0.052
ET	53 (46.9)	16		76	
**Luminal B**					
CT-ET	88 (57.5)	21	0.473	49	0.465
ET	65 (42.5)	19		58	
**Cohort II**					
**Luminal A**					
CT-ET	51 (38.9)	21	0.386	47	0.082
ET	80 (61.1)	21		57	
**Luminal B**					
CT-ET	121 (51.5)	16	0.208	45	0.093
ET	114 (48.5)	18		49	

CT = chemotherapy; ET = endocrine therapy; OS = overall survival; PFS = progression-free survival.

## Data Availability

The data presented in this study are available on request from the corresponding author. This published paper contains all of the data produced or analyzed during this investigation.
